# Two Cases from My Note-book*Read by title before the Southern Section of the American Otological, Rhinological and Laryngological Association, Atlanta, March 28, 1898.

**Published:** 1898-06

**Authors:** W. C. Galloway

**Affiliations:** Wilmington, N. C.


### TWO CASES FROM MY NOTE-BOOK.*

By W. C. GALLOWAY, M.D.,

Wilmington, N. C.

### FOLLICULOUS PHARYNGITIS.

A gentleman, school-teacher, twenty-two years old, of healthy ancestry, visited my office from the country, September 29, 1896, with the following history: Last Christmas, nine months ago, throat became sore and tonsils swelled and were painful. During childhood throat gave him a good deal of trouble, but got well and had not bothered him until last Christmas. The attack then, he thinks, was brought on by going to bed with cold feet after playing in the snow. Throat grew gradually worse; felt raw and like it was filled up. Hawking and coughing, which were moderate at first, became frequent and annoying and sometimes distressing, tough, glairy mucus being freely expectorated. As the disease progressed deglutition was troublesome and difficult. There was some dripping at the back of the throat, and the nose discharged more than usual. Voice husky and lacking in timbre; appetite failed, weight diminished; palms of the hands were moist, face pale, eyes lusterless, mind inactive; felt languid, no disposition to exercise; was nervous and fretful; nocturnal emissions frequent and depressing; and finally had to abandon his school. He was certainly a woebegone looking specimen, and not being whole, needed a physician. Came for treatment and wanted my advice about the propriety of his seeking a warmer southern climate, as though North Carolina were not a real, tangible Utopia between the first and third heavens, in a fertile valley, sheltered by mountains and fanned by the sea. His was the worst type of the disease I had seen, and I feared tubercular complications had already marked him for that undiscovered country. He was five feet eleven inches in height and did not weigh exceeding 110 pounds.

---

*Read by title before the Southern Section of the American Otological, Rhinological and Laryngological Association, Atlanta, March 28, 1898.

Examination revealed both tonsils considerably enlarged and diseased—folliculous tonsillitis, the twelve or fifteen orifices being filled with an offensive cheesy deposit. The pharyngeal wall was vascular, very much engorged, and studded with numerous hypertrophied follicles, some crowned with a spot of white and others filled with caseous material, which partly protruded out of the orifices of the crypts. They were as thick and shiny as white seed in the pulp of a red-meated melon. I make the comparison because it is true, and it has the merit of originality; and it is an opportune time to make the mouth begin to water in anticipation of the luscious Georgia rattlesnakes. The back of the tongue was typical of the pharynx, and so much swollen as largely to hide the epiglottis. Larynx and vocal cords somewhat congested from constant coughing, otherwise normal; and the nasal cavities were congested and slightly inflamed posteriorly. Having been a general practitioner for a number of years, I took the precaution to auscultate and percuss the lungs, but found them in good condition. Amputated both tonsils with Mathieu's tonsillotome, first applying a four per cent. cocain solution; hemorrhage insignificant. Waited a week and began treating nose and throat, using for the former the following:

| | | |
|---|---|---|
| ℞ | Zinci sulphocarbolate | gr. x |
| | Cocain muriat | gr. iv |
| | Borolyptol / Glycerin } *aa* | ʒ iv |
| | Aquæ menth. pip. q. s | ℥ iv |

M. Sig.: spray *ter in die.*

Same spray was also used for the throat, it being bland, antiseptic and healing. Touched a number of the follicles with the galvano-cautery and used argent. nitrat., 40 grs. to oz. of water, twice a week for awhile, and afterwards once weekly. Gave tonics, directed him to remain out of doors a good deal, a part of the time in the sunshine, take gentle exercise, and, as his appetite improved, to eat the best tissue-building food he could command.

He has steadily improved, but is not thoroughly well yet, though nearly so. Cough gone, pharynx about healed, tongue almost so, rhinitis cured, appetite vigorous, digestion good, sleeps well, emissions ceased, general health fine, weighs 150 pounds, looks almost ruddy, works ten or twelve hours a day as a stenographer—more than he ought to do—and is a good candidate for matrimony, a long life, and heaven.

### FOREIGN BODY IN THE LARYNX.

A white boy eleven years of age came to the office in great distress the night of July 31, 1897. Said while picking his teeth with a sandspur (a novel instrument for that purpose) it got caught between his teeth, and in an endeavor to extract it with a pin it suddenly became dislodged and fell into his windpipe. A feeling of suffocation immediately followed, he breathed with great difficulty, coughed violently, and whenever he coughed or made an effort to swallow the spur pricked him and gave him considerable pain. When he reached me two hours after the accident (he came by direction of his physician) his face wore an anxious expression, tears trickled from his eyes, and his breathing, though more quiet, was labored, and he could only talk in a whisper. Found the upper part of the larynx unduly red and congested, and the sandspur impinged upon and partly sticking into the vocal cords externally and at their anterior attachment, covering nearly one-half of the opening. Had it not been attached by one of the spears it doubtless would have gone farther down the larynx, or might have been expelled.

Applied a four per cent. cocain spray freely, but had some trouble in using the laryngeal forceps, due to retching and gagging, the larynx being peculiarly susceptible, but finally grasped it, and as I did so the patient vomited and I lost the spur, but extracted it from the larynx, and relief was instantaneous. The voice, however, was not immediately restored, due to the congestion of the cords and adjacent tissues. It gradually returned and by the next day he talked very well.

I think this is the first record of a sandspur lodging in the larynx, and only in that respect is the case novel.

---

Note.—The sandspur is what I take to be the fruit of a species of graminæ, which grows abundantly in this and possibly all warm sections near the coast, and during summer and fall is a terror to barefooted boys and hunting dogs.

